# Random vs. blocked perturbation training on reactive balance control in peripheral neuropathy: A protocol study for a randomized controlled trial

**DOI:** 10.1016/j.mex.2026.103796

**Published:** 2026-01-12

**Authors:** Razieh Javadian Kootenayi, Razieh Mofateh, Mehrnoosh Zakerkish, Neda Orakifar, Saeideh Monjezi, Mohammad Mehravar, Maryam Seyedtabib

**Affiliations:** a Rehabilitation Research Center, Ahvaz Jundishapur University of Medical Sciences, Ahvaz Postal code: 6135715794, Iran; b Diabetes Research Center, Health Research Institute, Ahvaz Jundishapur University of Medical Sciences, Ahvaz Postal code: 6135715794, Iran; c Department of Biostatistics and Epidemiology, School of Public Health, Ahvaz Jundishapur University of Medical Sciences, Ahvaz Postal code: 6137515751, Iran

**Keywords:** Diabetic neuropathy, Postural balance, Learning

## Abstract

Diabetic peripheral neuropathy (DPN) is the leading cause of disturbances in reactive balance control. The repeated, external mechanical perturbations in perturbation-based balance training(PBBT) evoke balance recovery strategies; which subsequently improve reactive balance performance. Using the practice schedule concept of motor learning in the design of PBBT is a relatively new approach related to balance exercises. This study aims to investigate the effects of blocked and random PBBT on reactive balance control and its persistency and transfer to conditions different from those experienced during training.

Individuals with DPN will be recruited and randomly allocated to one of the three groups: random, blocked, and control group. Random and blocked PBBT groups will receive single-session balance training, including unexpected perturbations of platform during quiet standing in two directions (anterior and posterior), and three difficulty levels of platform motion (displacement, velocity, and acceleration). Each balance perturbation in blocked group will be repeated over blocks of four trials. For the random group, perturbation sequence will be unpredictable for these four trials in each block. Primary outcomes (i.e., center of pressure variables, reaction time, movement time, and total response time variables) will be assessed at baseline as well as immediately and one day after intervention.

## Graphical abstract

**Figure:**
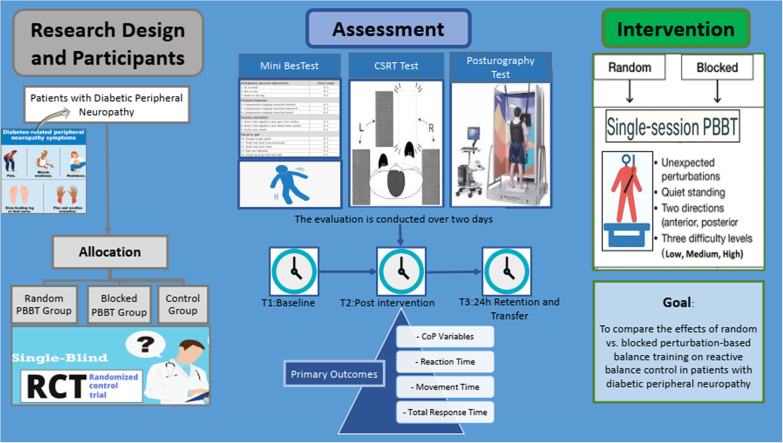

Specifications table

**Table:** 

Subject area	Neuroscience
**More specific subject area**	Learning
**Name of your protocol**	Random vs. Blocked Perturbation Training on Reactive Balance Control in Peripheral Neuropathy: Protocol Study for a Randomized Controlled Trial
**Reagents/tools**	- Participants' demographic information - Neuropathy Disability Score to assess the severity of neuropathy - Mini-Mental State Examination to test cognitive functioning - Short Beck Depression Inventory to assess depression in adolescents and adults -The Computerized Dynamic Posturography system [Smart EquiTest CRS (NeuroCom International Inc.Clockamas, Oregon)] to measure balance and postural control - long single force plate (EquiTest CRSVR NeuroComVR International, Clackamas, OR) and the screen to assess speed and coordination - Mini-balance evaluation systems test to evaluate dynamic balance
**Experimental design**	This is a protocol study for a single-blind randomized controlled trial. Individuals with DPN will be recruited and randomly allocated to one of the random, blocked, and control groups. Random and blocked PBBT groups will receive single-session PBBT involving platform perturbations during quiet standing in the anterior and posterior directions at three levels of difficulty. The control group will receive no intervention. All patients will be assessed at three time points (baseline, end of the session, and 24 hours later). Primary outcomes include center of pressure variables, reaction time, movement time, and total response time.
**Trial registration**	IRCT20240111060675N1
**Ethics**	- All individuals will provide written informed consent. - This study will be performed in line with the principles of the Declaration of Helsinki. Approval was granted by the Ethics Committee of Ahvaz Jundishapur University of Medical Science (approval date 2024-01-06/ number IR.AJUMS.REC.1402.537).
**Value of the Protocol**	- This study is the first to examine the effectiveness of PBBT using a blocked and random practice sequence on the acquisition, retention, and generalizability of reactive balance skills in individuals with DPN. - PBBT leads to faster balance adaptation and may potentially achieve better results with fewer sessions compared to traditional methods. - The results of this study will be helpful for the development of therapeutic exercises aimed at improving balance control and reducing the risk of falling in real-life challenges.

## Background

Diabetic peripheral neuropathy (DPN) affects over 50% of patients with type2 diabetes [1]. In these individuals, the degeneration of afferent and efferent nerve axons can lead to sensory loss, delayed reaction time, and muscle weakness, resulting in balance dysfunction and higher fall risk [2,3]. In daily life, falls mostly occur during dynamic activities due to an inability to control balance in response to mechanical perturbations caused by internal and external forces (impaired reactive balance control) [4]. Reactive balance control is one of the most important postural control strategies for preventing falls when the balance system is perturbed [5]. In individuals with DPN, damage to nerve fibers causes sensory impairment that slows the processing of sensory information and subsequent motor responses leading to a slower and less effective reactive balance response [6].

Research suggests that strategies to recover from unexpected loss of balance differ from voluntary exercises, indicating that fall prevention training should incorporate unexpected perturbations to increase the effectiveness of exercise [7]. Perturbation-based balance training (PBBT) is a form of reactive balance training that exposes individuals to repeated, external mechanical perturbations in a safe and controlled environment to elicit postural control reactions [8]. In the only study performed in individuals with DPN, a single PBBT session improved reactive balance, but the optimal sequence of perturbations for effective motor learning remains unknown [3].

Using the practice schedule concept of motor learning to PBBT is a relatively new approach [8]. There are two types of practice schedules:constant and variable. Constant practice involves repeatedly practicing a single skill. In contrast, variable practice exposes the learner to a variety of related skills [9]. Performing variable skills within a single practice session creates contextual interference (CI), a phenomenon that enhances long-term learning by requiring frequent shifts between task variations [10]. Variable practice is broadly categorized into blocked or random schedules [11]. Blocked practice, associated with low CI, involves repeated performing of one task before moving to another, facilitating skill acquisition and performance during training [11]. Random practice, associated with high CI, refers to practice task variations in the same session in a pseudo-random order [11]. Although acquisition may be slower, random practice leads to better skill retention and transfer to new situations [12].

Given the crucial role of reactive responses in preventing falls, which are often sudden and occur in any direction [13], it is important to investigate how different practice schedules affect the ability of individuals with DPN to react to balance perturbations. In addition, In individuals with DPN, compensating for sensory loss increases cognitive demand, causing balance impairments during dual-tasking [6]. Since reactive balance requires attention, dual tasks may further disrupt postural control strategies in these individuals [14]. Therefore, assessing the generalizability of improvements in reactive balance control during single-task to dual-tasks may be useful for enhancing dual-task interference on reactive balance performance in individuals with DPN.

To the best of our knowledge, no study has yet investigated the effects of different practice schedules of PBBT on reactive balance control in individuals with DPN. Therefore, this study aims to compare the effects of blocked and random practice of PBBT on the acquisition, retention, and generalizability of acquired reactive balance skills in individuals with DPN. Our findings would be helpful for the development of preventive and therapeutic strategies to reduce the risk of falls and improve reactive balance control in individuals with DPN.

## Description of protocol

### Study design

This single-blind randomized controlled trial will be conducted at the Rehabilitation Research Center located at the School of Rehabilitation Sciences, Ahvaz Jundishapur University of Medical Sciences, Ahvaz, Iran.

This study consists of two sessions. The first session consists of three parts; (1) pre-intervention assessments, baseline measurements will be performed by completing the questionnaires then implementing the pre-test, (2) 15-20 minutes of 24 unexpected perturbations while standing, and (3) post-test assessments. For the patients, this session will be followed by the second session 24 hours later, which include the retention and transfer tests. An overview of the study timeline, enrollment, and time points of assessment is presented in (Table 1).

**Table 1:** Participant flow of enrolment, assessments and interventions.

	T0	T1	TT	T2	T3
Recruitment	+				
Interview (inclusion/exclusion)	+				
Assigning Consent form		+			
Randomization		+			
Questionnaires (MMSE; NDS; BDI-13; Mini-BEST)		+			
Assessment of reactive balance responses (balance reactions while standing in pre-test, post-test, and retention test)		+		+	+
Perturbation training intervention			+		
Transfer tests		+		+	+
Dual task test transfer (Concurrent mental arithmetic task)		+			+
Rotational mode transfer		+		+	+
Choice Stepping Reaction Time (CSRT) Test		+		+	+

T0, prior to study; T1, baseline (pre-intervention); TT, training time; T2, post-intervention test; T3, second session (one day post-intervention). Abbreviations: MMSE, Mini-Mental Scale Examination; NDS, Neuropathy Disability Score; BDI-13, Short Beck Depression Inventory; Mini-BEST, Mini Balance Evaluation Systems Test.

### Inclusion and exclusion criteria

A total sample of 60 individuals with DPN and type2 diabetes aged between 45 and 60 years suffering from peripheral neuropathy (Neuropathy Disability Score: three to five), able to walk without assistive devices, having the ability to stand independently, obtaining a score above 24 on the Mini-Mental State Examination, and able to read and write the Persian language will be included in the study.

Exclusion criteria will be foot ulcer and amputation; orthopedic problems; severe pain in the soles of the feet; symptoms of central nervous system problems such as dementia, Parkinson’s disease, multiple sclerosis, and a history of stroke; uncorrected vision problems; hearing problems; a history of vertigo; suffering from peripheral vascular and heart diseases which limit exercise, such as uncontrolled blood pressure, unstable angina, and a history of resting tachycardia or arrhythmia; participation in professional or regular exercises for at least six months before the study; taking antipsychotic and anti-anxiety drugs; and depression (Short Beck Depression Inventory˃18).

The neuropathy Disability Score is a validated scoring tool used to investigate the severity of neuropathy, particularly in the extremities [15]. It evaluates vibration sense, temperature sense, pinprick sensation, and ankle reflex for quantifying the level of neurological impairments. The score ranges from 0 to 10, which scores of three to five indicate mild neuropathy [16]. The mini-Mental State Examination is a standardized questionnaire used to assess cognitive function and screen for cognitive disorders. The test consists of 11 questions, with a maximum score of 30. A score of 23 or below generally indicates cognitive impairment [17]. The short Beck Depression Inventory is designed for rapid screening and measuring of depression in adolescents and adults. A score greater than 18 indicates moderate to severe depression [18].

Patients will be referred and recruited from those visiting the diabetic department of hospitals and clinics, with an endocrinologist's approval. The outcome assessor, who will be unaware of group assignment and training, will examine the patients for the selection criteria before enrollment. This research will be conducted in accordance with the ethical recommendations of the Declaration of Helsinki. The informed consent statement will be signed by all subjects. After randomization, participants' demographic information, medical history, and prior falls will be documented.

### Random allocation

The stratified permuted block randomization method will be used to assign participants to one of the three groups: blocked PBBT, random PBBT, and the control group. It combines stratified randomization, which divides participants into two subgroups based on gender, with permuted block randomization, which uses blocks of treatment assignments to maintain balance within those subgroups. A random order of three letters, A (intervention with a random sequence), B (intervention with a blocked sequence), and C (control), will be made. The sequences will be put inside an opaque envelope.

### Interventions

For the intervention groups (random and blocked PBBT), the training will be performed by a different physical therapist. Patients in the control group will receive no intervention.

Participants will be stand on two force platforms of computerized dynamic posturography with one foot on each of the platforms. The intervention involves restoring body balance after unexpected perturbations through movement of the support base in the anteroposterior direction, for both directions at three levels of difficulty. Participants will be verbally instructed to maintain their body balance after support base displacements. Ground reaction forces underneath each leg will be recorded by two adjacently placed movable platforms of the computerized dynamic posturography device.

Participants will wear a safety harness supported by ropes tied at shoulder height, with the other end attached above their head. While these ropes will not limit balance recovery movements, they prevent participants from falling.

Researchers selected three difficulty levels for reactive balance training: low difficulty (3cm displacement amplitude; 5cm/s peak velocity and 70cm/s^2^ peak acceleration), medium difficulty (7/5cm displacement amplitude; 10cm/s peak velocity and 137cm/s^2^ peak acceleration), and high difficulty (15cm displacement amplitude; 15cm/s peak velocity and 260cm /s^2^ peak acceleration). From combining perturbation directions (anterior and posterior) and three difficulty levels of platform motion, six conditions of block perturbations will be generated.

In each block, participants will perform four trials for each perturbation condition, given by the combination of direction and difficulty levels, totaling 24 trials. In the blocked group, each balance perturbation will be repeated over blocks of four trials for participants. For the random group, the four perturbations of each block will be scheduled pseudorandomly and unpredictably, with no repetition of a perturbation condition (difficulty × direction) in consecutive trials.

The intervention groups will perform this single session PBBT after a five-minute rest at the end of the examining pre-intervention tests. The duration of each trial is 10 seconds and a 10 second rest is allowed between trials. In addition, a one-minute rest is given between blocks.

### Methods of measurement

Participants will be evaluated at a pre-test (T1), post-test (T2), and retention test (T3) using the same protocol. In these tests, each participant will receive two trials of perturbations with each of the three difficulty levels and the anterior direction with a serial sequence (totaling six trials). Serial sequence refers to the sequence or arrangement of different skills, or variations of a skill, in a specific order, one after another in a predictable manner. The post-test is applied five minutes after PBBT.

The retention test will be applied at the beginning of the second session. After a five-minute rest, patients will be tested in three different transfer tasks.

Transfer tasks will be assessed as follows:

Cognitive dual-tasking: counting backward by three from a random number selected between 100 and 200 by the outcome assessor during perturbations. Participants will be asked to maintain balance while they attempt to perform the consecutive mental subtractions task as quickly and as accurately as possible, speaking aloud the result. During this cognitive transfer task, the high level of difficulty perturbation will be applied in the anterior direction, with two trials.

Different mode of perturbation: In this test, the perturbation will be in the anterior (dorsiflexion) direction with a rotational mode, with two trials.

Choice Stepping Reaction Time (CSRT) Test: for assessing the transferability of improvements obtained in the reactive balance responses to the balance responses against internal perturbations, the CSRT test will be used. In this test, patients will stand on a long force plate of the Neurocom device with their feet hip-width apart, and while looking at the screen in front of them, colored boxes are randomly displayed on the right or left side of the screen. Participants will be instructed to step as fast as possible with the respective leg and then return to the starting position. Each participant will perform 10 trials, which include five repetitions for each direction randomly [19]. A one-minute rest period will be allowed between transfer tests. In order to have a baseline in this study, all transfer tests will be assessed before training.

Reactive balance control is the main outcome in this study, which will be recorded with the Computerized Dynamic Posturography system in the standing position [20].

Reactive response variables will be collected by the Smart EquiTest CRS (NeuroCom International Inc.Clockamas, Oregon) device, including: (1) peak center of pressure (CoP) displacement in anterior and posterior directions which indicates the maximum distance that the location of the CoP reaches in the anterior or posterior direction relative to the baseline [21]; (2) response time, defined as the time interval between the start of platform movement (as a stimulus) and the initiation of the participant's active response; (3) peak values of CoP velocity in the anterior and posterior directions following the onset of platform motion.

CSRT test is used to measure how quickly and accurately a person can step in response to a visual stimulus [22]. The equipment will be used consisted of the long single force plate (EquiTest CRSVR NeuroComVR International, Clackamas, OR) and the screen in front of patients.

In the step execution data, the following temporal variables will be examined: (1) Reaction time (RT) defined as the time interval between the appearance of the stimulus and the movement initiation (lifting the corresponding leg); (2) Movement time (MVT) defined as the time interval between the start of the movement and the foot strike; and (3) Total response time (TRT) calculated as the sum of RT and MVT [23].

In addition to other interventions, the Mini-Balance Evaluation Systems Test (Mini-BESTest) will be performed as a clinical assessment tool. This test evaluates dynamic balance, particularly in individuals with balance impairments [24].

### Statistics

All data will be analyzed using SPSS version 28. Baseline data and demographic characteristics will be described using descriptive statistics.

Data distribution normality will be confirmed using the Kolmogorov–Smirnov test. The main effects and interactions of group (blocked sequence, random sequence, and control), time (pre-test and post-test), and difficulty level (low, medium and high) on CoP variables will be compared using three-way repeated measures ANOVA. In addition, the main effects and interactions of groups, time (post-test and retention), and difficulty level (low, medium, and high) on CoP variables will be compared using three-way repeated measures ANOVA. Also, a one-Way ANOVA will be used to compare the means of CoP variables between the retention and transfer phases across three groups. Moreover, a two-way repeated measure ANOVA will be used to determine the main effects and interactions of group and time (pre-test, post-test, and transfer) on RT, MVT, and TRT variables of the CSRT test. A post-hoc Bonferroni test will be used for multiple comparisons.

### Sample size calculation

Due to a lack of similar studies, we conducted a pilot study on 30 individuals with diabetic neuropathy (10 for each group). Sample size was calculated in G*power software version 1.9.4.3 and was based on the primary outcome of the pilot study, the difference between means of the peak CoP velocity in the anterior direction measured with the computerized dynamic posturography at T1 and T2. Based on the statistical power of 80% and the probability of the first type error of 0.05, the sample size was estimated to be about 20 individuals in each group.

### Protocol validation

The clinical study corresponding to our protocol is still underway, with subject recruitment and intervention therapy currently in progress. As we have not yet reached the outcome assessment phase, data to validate the protocol have not been generated.

## Limitations

Several limitations must also be considered. Although a safety harness is used to protect patients from falls, there is a risk of balance loss and fall-related injury. Since perturbation training is a challenging training approach, some patients may discontinue participation in this program. Our study will address diabetic patients with mild severity of neuropathy; Therefore, the results cannot be generalized to diabetic patients with other severities.

## CRediT author statement

Razieh Javadian Kootenayi: Conceptualization, Methodology, Validation, Acquisition of data, Interpretation of data, Writing - original draft, Writing - review & editing. Razieh Mofateh: Conceptualization, Methodology, Validation, Interpretation of data, Writing - original draft, Writing - review & editing, Supervision, Project administration, Funding acquisition. Mehrnoosh Zakerkish: Conceptualization, Methodology, Interpretation of data, Writing - original draft, Writing - review & editing. Neda Orakifar: Conceptualization, Methodology, Interpretation of data, Writing - original draft, Writing - review & editing. Saeideh Monjezi: Conceptualization, Methodology, Interpretation of data, Writing - original draft, Writing - review & editing. Mohammad Mehravar: Conceptualization, Methodology, Interpretation of data, Software Writing - original draft, Writing - review & editing. Maryam Seyedtabib: Conceptualization, Methodology, Statistical analysis, Writing - original draft, Writing - review & editing.

## Declaration of competing interest

The authors declare that they have no known competing financial interests or personal relationships that could have appeared to influence the work reported in this paper.

## Data Availability

No data was used for the research described in the article.
